# Across-environment seed protein stability and genetic architecture of seed components in soybean

**DOI:** 10.1038/s41598-024-67035-4

**Published:** 2024-07-16

**Authors:** Chengjun Wu, Andrea Acuña, Liliana Florez-Palacios, Derrick Harrison, Daniel Rogers, Leandro Mozzoni, Rouf Mian, Caio Canella Vieira

**Affiliations:** 1 Department of Crop, Soil, and Environmental Sciences, University of Arkansas, Fayetteville, AR 72701 USA; 2 Soybean and Nitrogen Fixation Research Unit, USDA-Agricultural Research Service, Raleigh, NC 27607 USA

**Keywords:** Plant breeding, Plant genetics

## Abstract

The recent surge in the plant-based protein market has resulted in high demands for soybean genotypes with improved grain yield, seed protein and oil content, and essential amino acids (EAAs). Given the quantitative nature of these traits, complex interactions among seed components, as well as between seed components and environmental factors and management practices, add complexity to the development of desired genotypes. In this study, the across-environment seed protein stability of 449 genetically diverse plant introductions was assessed, revealing that genotypes may display varying sensitivities to such environmental stimuli. The EAAs valine, phenylalanine, and threonine showed the highest variable importance toward the variation in stability, while both seed protein and oil contents were among the explanatory variables with the lowest importance. In addition, 56 single nucleotide polymorphism (SNP) markers were significantly associated with various seed components. Despite the strong phenotypic Pearson’s correlation observed among most seed components, many independent genomic regions associated with one or few seed components were identified. These findings provide insights for improving the seed concentration of specific EAAs and reducing the negative correlation between seed protein and oil contents.

## Introduction

Soybean [*Glycine max* (L.) Merr.] produces the highest amount of protein per hectare and accounts for over 60% of total global oilseed production, thus being a crop with versatile uses from a primary source of plant-based protein in human food and animal feed to a renewable alternative to petroleum diesel^1,2^. The United States is expected to produce nearly 117 million Mt from 33.6 million hectares in 2023, representing 29% of global soybean production^2^. After processing the grain, the valuable products derived from soybean are protein meal and oil. Soybean meal is primarily used as a source of protein and amino acids in cattle, swine, and poultry feed. Global soybean meal production exceeds 270 million Mt^2^. The United States accounts for 19% of global soybean meal production (51.7 million Mt) and 21% of global exports (15.7 million Mt)^2^. In the United States, nearly 50% of soybean meal consumption is attributed to broilers (15.8 million tons), followed by hogs (23%, 7.5 million tons) and egg-laying hens (9.3%, 3.0 million tons)^3^. Because of the high quality and seed content of protein and amino acids relative to other widely cultivated row crops, soybean is the major source of plant-based protein in livestock rations^4^.

Proteins are a vital part of the diet of humans and animals as they are the source of balanced amino acids which are the functional subunits of proteins that are intermediates for many biosynthesis pathways^5^. Based on nutritional requirements, amino acids are divided into three types: essential amino acids (EAAs), semi-essential amino acids (SEAAs), and non-essential amino acids (NEAAs). Essential amino acids are not synthesized by either humans or animals but are extremely indispensable for various biosynthesis pathways and must be consumed in the diet. Semi-essential amino acids are synthesized in the body, but the amount produced is insufficient to properly complete various biosynthesis pathways. Non-essential amino acids are produced and stored by either humans or animals in sufficient amounts^6^.

Soybean seeds contain all eight standard EAAs for human nutrition including isoleucine, leucine, lysine, methionine, phenylalanine, threonine, tryptophan, and valine^7^. In addition, glycine (needed for poultry feed) and arginine (needed for poultry, swine, and fish feed) are present in soybean seeds^8^. However, the ratio of amino acids is not always desirable for feed rations, and the amino acid composition of soybean meal could be a limiting factor in the linear programming of feed ratio formulations^9–11^. For instance, soybean seed concentrations of sulfur-containing amino acids cysteine and methionine are relatively lower than desired levels for the optimum growth of monogastric animals such as poultry and swine by 2.0 and 3.0%, respectively^12^. Soybean meal protein is also deficient in the EAAs threonine and lysine as the sole dietary source of protein for animal feed, and a 10% increase in the concentration of these amino acids would improve the commercial value of soybean meal^13^.

Given that seed protein and oil content and amino acids (EAAs, SEAAs, and NEAAs) composition are key factors in determining soybean seed quality for feed and food uses, it is important to optimize the seed composition and ratio of amino acids in soybean cultivars^14–17^. Currently, seed protein content in soybean cultivars ranges between 330 and 390 g kg^−1^ at 130 g kg^−1^ moisture content^18^. Historical breeding efforts for soybean cultivars with higher seed oil content and yield potential have negatively impacted seed protein content^19,20^. Therefore, the development and identification of cultivars and germplasm with high seed protein content and appropriate ratio of amino acid concentration may play an important role to supply plant-based protein for human and animal nutrition.

Several genomic regions associated with seed protein as well as amino acid composition in soybean have been reported. According to Soybase.org, there are currently over 250 reported quantitative trait loci (QTLs) associated with soybean seed protein content, and a much lower number of QTLs and genomic regions associated with various amino acids (www.soybase.org). The first report of QTLs associated with soybean seed protein dates back more than 30 years when Diers et al. (1992) reported significant associations on chromosomes 20 (linkage group (LG) I) and 15 (LG E)^21^. Subsequent studies have reported and confirmed the genomic regions identified on chromosomes 20 and 15 using populations with various genetic backgrounds, tested in diverse environments, and genotyped with distinct molecular markers density^22–28^.

Although genetics account for a larger portion of the phenotypic variation in soybean seed protein content compared to the environment, genotype by environment interaction (G×E) significantly affects seed protein and amino acid composition in soybean^29,30^. Various environmental covariates affect soybean seed protein content and composition, including temperature^31–35^, water stress^36,37^, and nitrogen availability^36–38^. However, the rationale behind genotypes showing variable across-environment seed protein stability under different environmental conditions is still unknown. To date, limited studies have investigated the effect of various seed components (seed protein and oil contents, and EAAs concentration) on seed protein stability across diverse environments. Therefore, the goals of this study were to i) characterize the genetic architecture of seed protein and oil contents, as well as EAAs concentration in a panel of genetically diverse soybean accessions and ii) identify seed components that are contributing to the across-environment seed protein stability.

## Material and methods

### Plant materials and data collection

A total of 449 genetically diverse plant introductions (PIs) ranging from maturity group (MG) 4 to 6 were used in this study. The distribution of PIs based on maturity consisted of 126 MG 4 PIs, 181 MG 5 PIs, and 142 MG 6 PIs. Accessions were obtained from the USDA Soybean Germplasm Collection and originated from 16 countries, including South Korea (173), Japan (116), United States (67), China (59), North Korea (11), Argentina (3), Mexico (3), one accession from India, Morocco, Pakistan, South Africa, Turkey, Uganda, Vietnam, and Zimbabwe, respectively, and nine accessions with an unknown origin. All entries were genotyped with the SoySNP50K BeadChip^39^ and data has been made available by authors at the SoyBase Genetics and Genomics Database (http://soybase.org/snps/download.php)^40^. Non-polymorphic single nucleotide polymorphisms (SNPs) were removed, resulting in 41,080 SNPs being used in the genome-wide association studies (GWAS). We confirm that our field studies involving plants, whether cultivated or wild, adhered to applicable institutional, national, and international guidelines and legislation.

Field trials were conducted in seven environments (unique combination of location and year) at the Milo J. Shult Agricultural Research and Extension Center in Fayetteville, AR in 2017 and 2018 (36°05′56′′ N, 94°10′42′′ W), the Northeast Research & Extension Center in Keiser, AR in 2017 and 2018 (35°40′27’’N, 90°05′15′′ W), the Rice Research & Extension Center in Stuttgart, AR in 2017 (34°28′31′′ N, 91°25′07′′ W), and the Rohwer Research Station in Rohwer, AR in 2017 and 2018 (33°48′35′′ N, 91°16′13′′ W). Trials were conducted using a randomized design. Each plot consisted of one 3-m long row with 0.75 m row space and approximately 100 seeds were sowed per plot. At physiological maturity^41^, seeds were hand-harvested. A 20-g seed sample per genotype per environment was used for near-infrared reflectance spectroscopy (NIR) analysis using an IM 9500 Grain Analyzer (Perten Instruments®, Hägersten, Sweden) to determine seed protein and oil content, and EAAs concentration. The high protein cultivar ‘Osage’^42^ was used as the reference check. Multi-environmental data (dry weight basis) was analyzed in R^43^. Adjusted means across environments were calculated utilizing the package ‘*lmerTest*’^44^ based on a mixed-effects linear model fitted using the package ‘*lme4*’^45^. The model included the fixed effect of genotype and the random effect of the environment (location × year).

To measure the reliability and consistency of the collected phenotype, Cronbach’s alpha (α) score^46^ was calculated for each trait using the R package ‘*psych’*^47^ following Eq. (1). To understand the inter-relatedness between seed components, Pearson’s correlation coefficients were calculated between all possible pairs of traits based on the adjusted means utilizing the function ‘*cor*’ in R.

1 $$\alpha = \frac{{k \, {\times} \,  \overline{c}}}{{\begin{array}{*{20}c} {\overline{v} + \left( {k - 1} \right) \overline{c}} \\ \end{array} }}$$

where *k* is the number of observations per seed component, *c* is the average inter-item covariance of observed seed components between each pair of environments averaged for all pairs of environments, and *v* is the average variance of each observed seed component across all environments.

### Seed components effect on across-environment seed protein stability

To obtain the across-environment seed protein stability, Shukla’s Stability Variance (*σi*^2^)^48^ was calculated using the R package ‘*statgenG*×*E*’^49^. Shukla’s stability refers to the variance around the genotype’s phenotypic mean across all environments. It partitions the total G×E term into unbiased estimates of the G×E variance attributable to each genotype which provides a measure of stability without accounting for the performance of the genotype^50^. Shukla’s Stability Variance considers a genotype stable if its response to multiple environments is parallel to the mean response of all genotypes in the test^51^.

To determine the effect of seed components on seed protein stability (metric derived from Shukla’s Stability Variance), the variable importance was calculated based on the “Gini Importance” obtained from a Random Forest model (RF)^52^. RF is a supervised learning algorithm based on the assembly of multiple decision trees which conducts feature selection and generates non-correlated decision trees^52^. In summary, the Gini Importance indicates how often a variable is selected for a given split across all trees and the magnitude of its discriminative value (decrease in node impurity) for the classification problem^52,53^. The ‘Gini Importance’ for each seed component was retrieved using the function ‘*importance*’ of the R package *‘caret’*^54^. The model was fitted using Shukla’s Stability Variance as the variable response and the EAAs concentration as explanatory variables utilizing the function ‘*train’* of the R package ‘*caret*’ with the method set to ‘*rf*’. The dataset was divided into training and testing sets consisting of 70 and 30% of the observations, respectively. The model was tuned by going through a grid search for the hyperparameter *‘mtry’* (1–5, number of variables randomly collected to be sampled at each split time), with ‘*ntree*’ set at 1000 (number of trees per aggregation) with ten-fold cross-validation. Both response and explanatory variables were standardized into z-scores (observed value subtracted by the sample average, and divided by the sample standard deviation) utilizing the function ‘*scale*’ in R.

### Genetic architecture of seed components

To unveil the genetic architecture of seed protein and oil contents, as well as EAAs concentration, genome-wide association studies (GWAS) were conducted to detect significant marker-trait associations between SNPs and each seed component. The Bayesian-information and Linkage-disequilibrium Iteratively Nested Keyway (BLINK)^55^ model was applied to conduct GWAS using the R package “*GAPIT*”^56^. The adjusted means across environments for each trait were used as the variable response in each model. In essence, BLINK represents an enhanced version of the Fixed and Random Model Circulating Probability Unification (FarmCPU)^57^. FarmCPU leverages the benefits of both mixed linear models and stepwise regression through their iterative use. It replaces kinship with a set of molecular markers, treated as fixed effects, which are individually tested at each iteration across the genome. These markers are optimized using a restricted maximum likelihood approach within a mixed linear model framework. This approach utilizes the variance and covariance established by the pre-selected markers, limiting the risk of model overfitting^57^. BLINK discards the assumption of even gene distribution related to a trait throughout the genome. It replaces the restricted maximum likelihood with Bayesian Information Content (BIC), leading to enhanced computational speed and efficiency^55^.

## Results

### Phenotype consistency and reliability across environments

Across all environments, both seed protein and oil contents have shown high Cronbach’s alpha (α) scores with confidence intervals ranging from 0.89 to 0.92 and 0.90 to 0.92, respectively, and minimum estimated error variance (0.17 and 0.15, respectively) (Table 1). Across genotypes, seed protein content averaged 440 g kg^−1^ with a standard deviation (SD) of 15 g kg^−1^; seed oil content averaged 190 g kg^−1^ with an SD of 10 g kg^−1^. Overall, EAAs have shown high reliability and consistency across environments with α scores ranging from 0.82 to 0.92 (Table 1). Tryptophan was the EAA with lowest α scores (confidence interval ranging from 0.55 to 0.66), although the obtained values are still within the acceptable consistent range^58–61^. Cronbach’s alpha scores can be interpreted as the correlation of the test with itself, of which the error variance is obtained by subtracting the squared α from 1.00^62^. In plant breeding, α scores can indicate the experimental quality and reliability of measured phenotypes. Like heritability, it can be explored as a measurement of the influence of genetic vs. nongenetic^63^.

**Table 1:** Summary of Cronbach’s alpha (α) scores and trait metrics across environments.

Trait	Cronbach’s α (CI 95%)^a^	Error^b^	Correlation^c^	Mean^d^ g kg^−1^	SD^e^ g kg^−1^
Seed Protein	0.89–0.92	0.17	0.57	440.0	15
Seed Oil	0.90–0.92	0.15	0.58	190.0	10
Isoleucine	0.88–0.91	0.19	0.53	20.0	0.6
Leucine	0.89–0.92	0.17	0.57	33.0	1.1
Lysine	0.89–0.92	0.17	0.56	28.0	0.8
Methionine	0.82–0.86	0.23	0.49	6.6	0.2
Phenylalanine	0.86–0.90	0.21	0.50	22.0	0.7
Threonine	0.87–0.90	0.21	0.51	16.0	0.5
Tryptophan	0.55–0.66	0.51	0.23	4.5	0.1
Valine	0.88–0.91	0.17	0.56	21.0	0.7

^a^Confidence interval (95%) of standardized α score.

^b^Estimated error variance was obtained by subtracting the squared α from 1.00.

^c^Inter-item average Pearson’s correlation.

^d^Average of each trait across all observed entries reported on dry weight basis.

^e^Standard Deviation of each trait across all observed entries reported on dry weight basis.

### Pearson’s correlation among seed components

**Figure 1:**
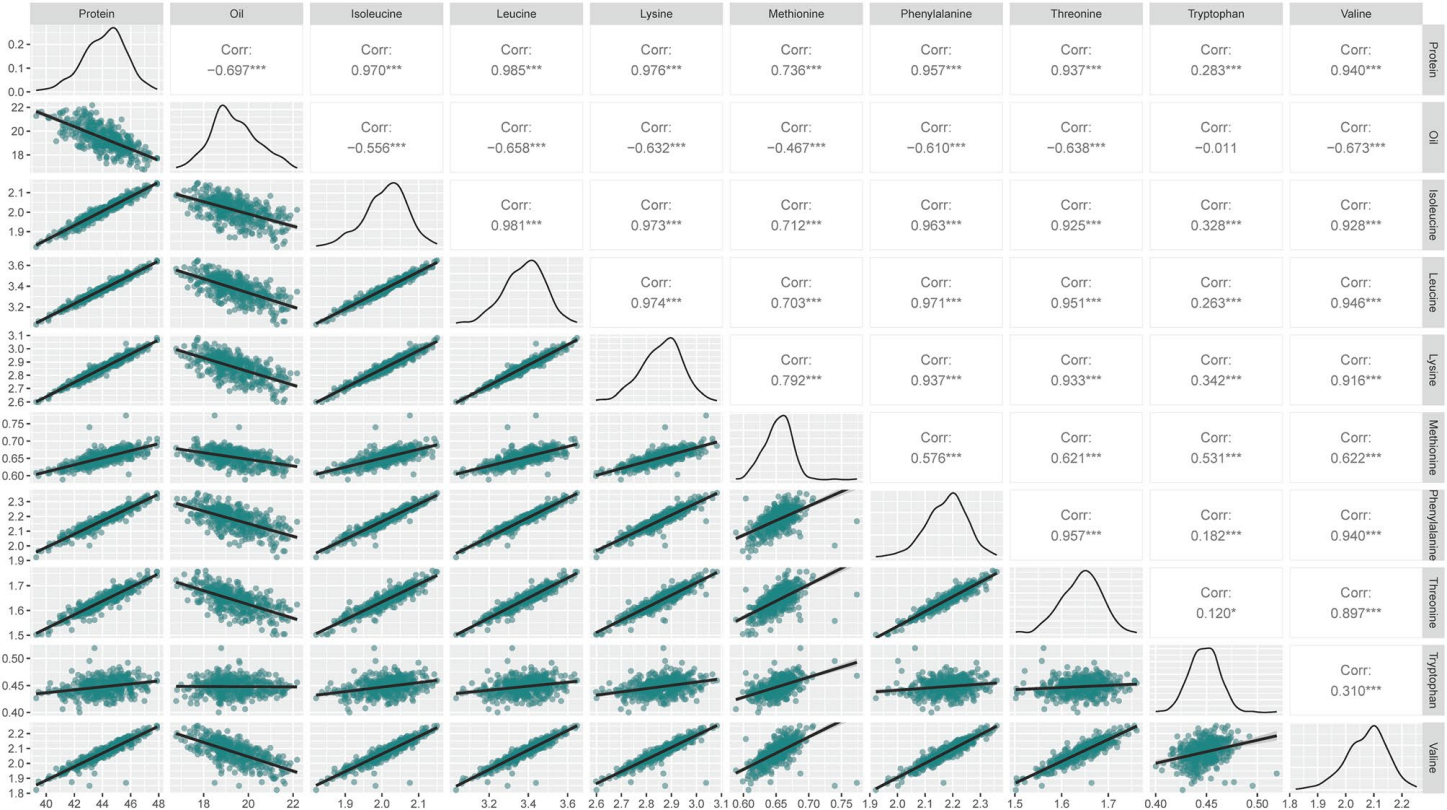
Phenotypic distribution of seed components and Pearson’s correlation among traits.

A normal distribution was observed for each seed-related trait confirming the availability of substantial phenotypic variance among genotypes (Fig. 1). Across genotypes, seed protein content was significantly correlated with seed oil content (− 0.697) and EAAs (0.283–0.985) (Fig. 1). As expected, there was a strong negative correlation between seed protein and oil contents, probably due to closely linked and/or pleiotropic loci regulating the traits’ expression. Although seed protein content was significantly correlated with all EAAs, methionine (0.736) and tryptophan (0.283) showed a lower correlation as compared to other EAAs (Fig. 1). Seed oil content, on the other hand, showed significant negative correlations with all EAAs (− 0.673 to − 0.467), except tryptophan (− 0.011). Interestingly, the strength of correlations between seed oil content and EAAs was not proportional to the strength of correlation between seed protein and oil. This indicates that investigating the composition of EAAs may be an interesting approach for increasing overall seed protein content, as well as reducing the degree of negative correlation between seed protein and oil. In addition, although the strength of the correlation between seed protein content and EAAs was similar, the correlation among EAAs does not follow a similar strength pattern indicating that it may be possible to modify the composition of soybean meal by manipulating individual EAAs through genetic recombination.

### Seed protein stability across environments

Across genotypes, Shukla’s Stability Variance ranged from 0.12 (PI 398351) to 17.16 (FC 33243), with an average of 2.70 (Supplementary Table 1). The 10 genotypes with the lowest Shukla’s Stability Variance and highest across-environment seed protein stability were PI 398351 (0.12), PI 424535A (0.14), PI 594567B (0.17), PI 398417 (0.19), PI 507068 (0.20), PI 507343 (0.20), PI 398399 (0.21), PI 398900 (0.23), PI 548586 (0.28), and PI 567791 (0.29). The 10 genotypes with the highest Shukla’s Stability Variance and lowest seed protein stability across environments were PI 574483 (8.85), PI 417070 (9.24), PI 181559 (9.88), PI 559370 (9.97), PI 408339 (10.55), PI 408337 (10.72), PI 221973 (10.80), PI 606749 (12.01), PI 548546 (12.27), and FC 33243 (17.16). The complete list of tested entries and respective Shukla’s Stability Variance is available in Supplementary Table 1.

**Figure 2:**
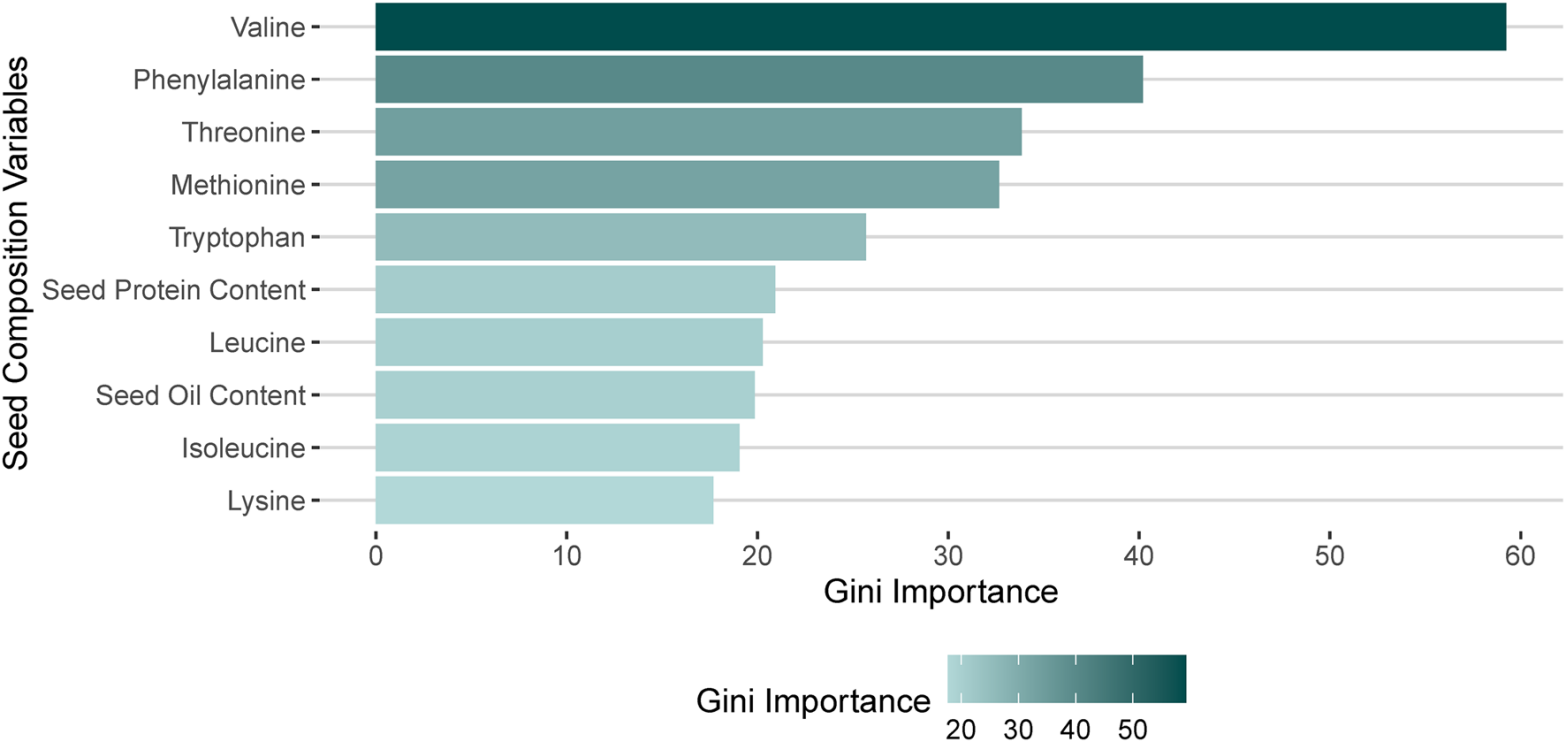
Gini Importance of seed components toward the variation of across-environment seed protein stability.

To understand the influence of seed components (seed protein and oil contents, and EAAs concentration) on the variation of seed protein stability across environments, the variable importance of each seed component was calculated based on the “Gini Importance” obtained from a Random Forest model (RF). The importance ranged from 17.68 (Lysine) to 59.24 (Valine), with an average of 28.94 (Fig. 2). Overall, the EAAs valine (59.24), phenylalanine (40.19), and threonine (33.84) were the variables with the highest importance, whereas lysine (17.69), isoleucine (19.06), seed oil content (19.85), leucine (20.27), seed protein content (20.93), and tryptophan (25.68) were the variables with the lowest importance in seed protein stability (Fig. 2). In general, seed oil content, methionine, and tryptophan were inversely proportional to across-environment seed protein stability, while seed protein content, isoleucine, leucine, lysine, phenylalanine, threonine, and valine were directly proportional to across-environment seed protein stability (Figure S1).

### Genetic architecture of seed components

**Figure 3:**
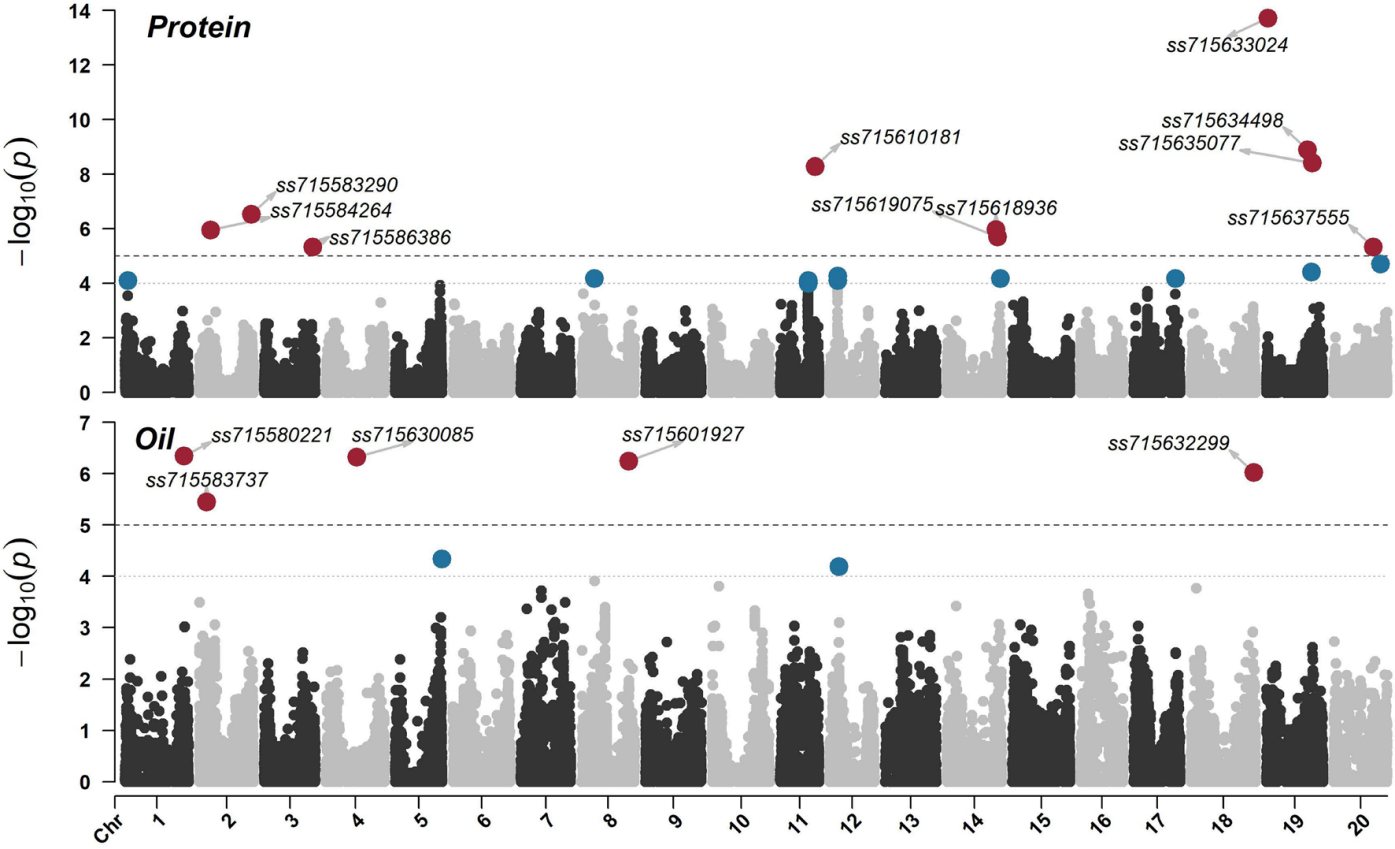
Manhattan plot of marker-trait associations for seed protein and oil contents.

A total of 56 SNPs across 19 chromosomes were significantly associated with one or more seed composition traits including seed protein (10 SNPs) and oil (five SNPs) content, as well as isoleucine (nine SNPs), leucine (one SNP), lysine (13 SNPs), methionine (13 SNPs), phenylamine (four SNPs), threonine (four SNPs), tryptophan (five SNPs), and valine (six SNPs) (Table 2). For seed protein content, marker-trait associations were distributed across chromosomes 2 (LG D1b), 3 (LG N), 11 (LG B1), 14 (LG B2), 19 (LG L), and 20, with the logarithm of odds (LOD) ranging from 5.3 to 13.7 (Table 2). The most significant marker-trait associations were detected on chromosome 19 and included the SNPs *ss715633024* (located at 1,066,013 bp, LOD of 13.7), *ss715634498* (located at 36,710,312 bp, LOD of 8.9), and *ss715635077* (located at 41,071,216 bp, LOD of 8.4) (Fig. 3). Interestingly, although seed protein and oil content were strongly correlated, the marker-trait associations for seed oil were in distinct genomic regions across five chromosomes including chromosomes 1 (LG D1a), 2, 4 (LG C1), 8 (LG A2) and 18 (LG G) (Table 2). The LOD scores ranged from 5.4 (*ss715583737* located at 6,494,883 bp of chromosome 2) to 6.3 (*ss715580221* located at 52,272,035 bp of chromosome 1) (Fig. 3). In addition, none of these five loci were significantly associated with any of the eight EAAs.

**Table 2:** Summary of significant marker-trait associations for seed protein and oil contents, as well as essential amino acids concentration.

SNP	Chr^1^	Position	Pro^2^	Oil^2^	Iso^3^	Leu^3^	Lys^3^	Met^3^	Phe^3^	Thr^3^	Try^3^	Val^3^
		bp	Logarithm of Odds (LOD)									
*ss715580221*	1	52,272,035	0.9	**6.3**^**4**^	0.1	0.1	0.3	1.3	0.0	0.3	0.0	0.4
*ss715583737*	2	6,494,883	1.5	**5.4**	0.8	2.8	2.9	0.7	0.9	0.1	0.1	1.4
*ss715583986*		8,317,216	1.7	2.2	3.6	2.1	0.1	2.2	**5.3**	0.2	1.0	1.4
*ss715584264*		9,821,649	**6.0**	0.6	1.0	0.2	2.3	2.1	1.0	0.1	0.1	0.8
*ss715583286*		46,284,615	0.2	0.8	2.8	1.7	**6.3**	0.4	1.3	4.0	0.3	1.5
*ss715583290*		46,430,339	**6.5**	1.1	3.1	1.8	0.5	0.4	1.3	0.0	0.3	1.6
*ss715586782*	3	520,176	1.0	0.5	1.7	0.7	2.9	**10.3**	1.3	0.8	0.2	0.9
*ss715586386*		43,147,264	**5.3**	0.3	1.1	3.6	**7.9**	2.5	3.6	2.6	0.7	4.8
*ss715630085*	4	27,452,088	0.6	**6.3**	0.0	0.5	1.6	1.0	0.9	1.0	0.4	0.5
*ss715591893*	5	40,302,839	3.9	1.6	**5.2**	2.4	**8.4**	0.2	**6.6**	4.0	0.2	3.3
*ss715591830*		40,695,722	2.4	1.6	0.3	0.1	0.5	**10.5**	0.5	0.7	0.2	0.6
*ss715593708*	6	17,265,495	0.7	0.2	1.2	1.0	1.3	**6.2**	0.9	1.0	1.7	1.6
*ss715594995*		48,191,362	1.5	2.9	3.2	0.9	1.1	**7.5**	1.4	2.5	0.2	2.2
*ss715595101*		49,712,499	0.4	0.7	0.3	0.1	0.6	0.8	0.0	0.0	**6.2**	0.3
*ss715598419*	7	5,982,921	1.4	0.0	0.5	1.3	0.2	0.5	0.5	0.5	0.1	**6.5**
*ss715597038*		28,982,904	1.1	1.0	0.7	1.6	0.6	**7.1**	0.5	0.4	0.0	0.6
*ss715598089*		42,222,270	1.1	0.5	2.2	1.4	1.6	**5.6**	1.5	0.9	0.7	2.2
*ss715601587*	8	3,756,587	2.5	0.0	2.0	0.6	**7.6**	2.2	1.2	0.3	0.0	0.3
*ss715599717*		15,577,877	0.4	0.4	**5.0**	2.1	0.9	0.9	1.5	0.6	1.8	0.1
*ss715601906*		41,920,703	1.0	0.1	0.4	0.3	0.8	0.3	0.4	**5.8**	0.1	1.4
*ss715601927*		42,090,911	0.8	**6.2**	0.2	3.7	1.0	0.4	4.7	0.3	0.1	1.5
*ss715604469*	9	43,968,962	1.5	0.4	1.0	0.5	1.5	**5.5**	0.0	0.3	1.7	0.1
*ss715606644*	10	38,659,084	1.4	0.8	**6.8**	1.8	1.2	0.6	2.3	0.9	1.5	0.7
*ss715607575*		46,498,104	0.8	0.2	**5.4**	3.2	0.6	1.0	1.7	0.1	0.5	0.2
*ss715611278*	11	9,083,067	1.3	0.3	**5.2**	1.9	1.2	0.6	1.7	2.4	0.8	1.2
*ss715609437*		27,119,917	2.0	1.1	1.4	2.6	**5.4**	1.3	2.5	2.0	0.3	0.3
*ss715610181*		31,076,850	**8.3**	1.9	**5.2**	3.1	**8.4**	1.7	2.0	4.9	0.1	0.0
*ss715617029*	13	14,949,741	1.9	0.1	2.2	1.3	**6.7**	0.6	2.4	1.4	0.7	0.7
*ss715616897*		15,547,215	0.5	0.4	0.3	0.0	0.5	**12.7**	0.7	0.1	3.0	1.0
*ss715614962*		30,325,993	3.0	1.7	2.3	1.0	2.1	0.9	1.4	1.6	0.3	**7.0**
*ss715615305*		32,525,645	1.4	0.2	1.3	2.6	1.0	1.2	2.1	0.8	0.0	**5.0**
*ss715618478*	14	3,556,174	0.5	0.3	0.6	0.4	0.4	0.4	0.6	0.0	**8.1**	1.2
*ss715618933*		43,746,289	0.7	1.3	0.3	3.9	1.3	1.0	3.9	**5.5**	0.4	2.2
*ss715618936*		43,763,886	**6.0**	0.2	**5.8**	4.3	1.7	1.1	3.8	2.5	0.1	1.6
*ss715619068*		45,179,507	0.2	0.0	2.2	2.0	**7.7**	1.1	1.8	0.9	1.7	0.3
*ss715619075*		45,198,143	**5.7**	0.4	2.8	3.2	1.4	1.1	2.5	3.4	1.1	0.0
*ss715623187*	15	9,067,087	0.7	0.2	0.2	0.4	0.2	**7.8**	0.7	0.2	0.7	0.2
*ss715623253*		9,591,733	2.9	1.2	**5.8**	3.1	2.4	1.4	1.8	1.2	0.0	3.3
*ss715620803*		14,594,541	1.0	0.1	0.6	0.1	2.1	**7.1**	0.3	0.2	1.3	0.3
*ss715621013*		15,735,835	0.4	0.2	0.4	0.2	0.0	0.8	0.0	0.3	**5.6**	0.2
*ss715622402*		48,841,245	2.4	0.9	1.3	2.0	2.2	0.0	1.6	3.5	0.2	**6.8**
*ss715623426*	16	133,118	1.2	0.0	1.1	1.5	0.9	0.4	0.8	0.6	**7.5**	0.1
*ss715624728*		35,145,590	0.7	0.1	1.7	1.4	2.0	**5.6**	0.5	0.3	0.7	0.7
*ss715626789*	17	3,399,801	1.1	0.5	1.0	0.8	2.5	**8.5**	0.4	0.8	1.0	0.7
*ss715632299*	18	56,161,047	0.2	**6.0**	0.3	0.4	1.4	0.2	0.1	0.4	0.6	0.0
*ss715633024*	19	1,066,013	**13.7**	0.8	2.4	**6.1**	**10.5**	3.3	1.9	**8.4**	0.2	**7.9**
*ss715634498*		36,710,312	**8.9**	0.7	4.0	1.7	2.4	0.8	2.8	3.2	1.0	4.1
*ss715634697*		38,142,874	0.1	0.5	0.2	3.3	1.4	0.6	**7.0**	0.9	0.8	0.1
*ss715634720*		38,372,492	2.1	0.7	**5.2**	0.4	2.6	0.4	1.7	1.0	1.5	**7.2**
*ss715634950*		40,180,166	2.0	0.5	1.2	1.0	**5.3**	1.1	1.3	1.1	1.4	0.7
*ss715635077*		41,071,216	**8.4**	0.5	4.0	1.1	**5.5**	0.8	3.9	**5.4**	1.4	0.5
*ss715635519*		45,628,241	2.3	0.3	0.8	0.6	1.5	**5.0**	0.2	0.4	1.7	0.1
*ss715636832*	20	10,496,202	0.1	0.1	0.0	0.3	0.2	0.9	0.2	0.0	**5.7**	0.3
*ss715637555*		35,363,119	**5.3**	2.3	1.9	4.4	**5.7**	0.4	2.1	3.0	0.1	3.7
*ss715638104*		40,704,783	1.7	0.9	3.7	2.3	**7.4**	1.9	**6.2**	4.1	0.7	0.8

^1^Chr, Chromosome. ^2^Pro, seed protein content; Oil, seed oil content.^3^Iso, isoleucine; Leu, leucine; Lys, lysine; Met, methionine, Phe, phenylalanine, Thr, threonine, Try, tryptophan; Val, valine. ^4^Values in bold are considered statistically significant (threshold of LOD > 5.0).

**Figure 4:**
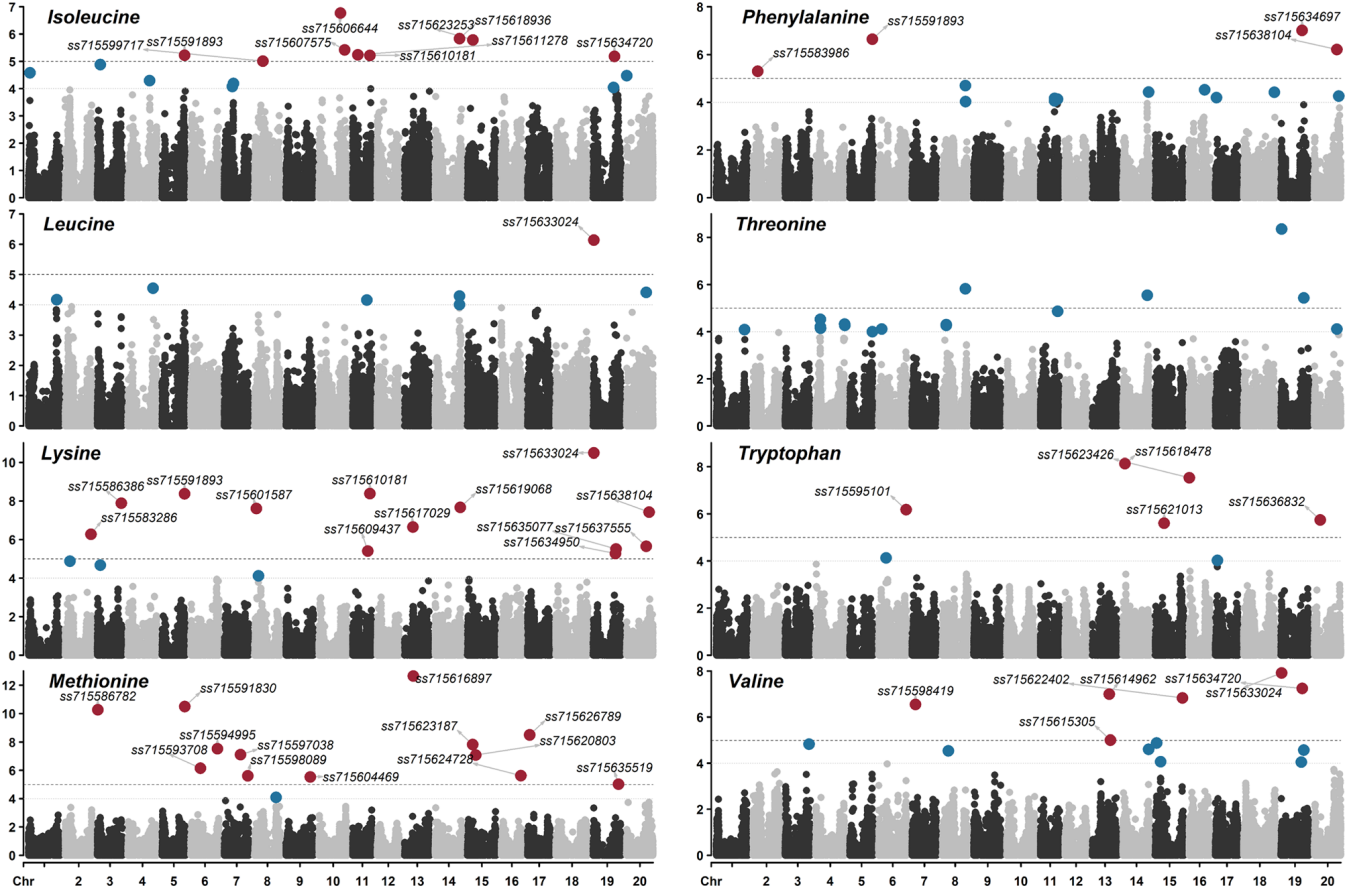
Manhattan plot of marker-trait associations for essential amino acids (EAAs) including isoleucine, leucine, lysine, methionine, phenylalanine, threonine, tryptophan, and valine.

Isoleucine had nine marker-trait associations detected across chromosomes 5 (LG A1), 8 (LG A2), 10 (LG O), 11, 14, 15, and 19, with LOD ranging from 5.0 (*ss715599717*, located at 15,577,877 bp of chromosome 8) to 6.8 (*ss715606644*, located at 38,659,084 bp of chromosome 10) (Fig. 4). The only significant marker-trait association detected for leucine was *ss715633024* (1,066,013 bp of chromosome 19), the same SNP that showed significant associations with seed protein content (LOD of 13.7), lysine (LOD of 10.5), threonine (LOD of 8.4), and valine (LOD of 7.9) (Table 2). A total of 13 significant marker-trait associations were detected for lysine across chromosomes 2, 3, 5, 8, 11, 13 (LG F), 14, 19, and 20 (Fig. 4). LOD ranged from 5.3 (*ss715634950*, 40,180,166 bp of chromosome 19) to 10.5 (*ss715633024*). For methionine, 13 marker-trait associations were detected across chromosomes 3, 5, 6 (LG C2), 7 (LG M), 9 (LG K), 13, 15, 16 (LG J), 17 (LG D2), and 19 (Fig. 4). LOD ranged from 5.0 (*ss715635519* located at 45,628,241 of chromosome 19) to 12.7 (*ss715616897* located at 15,547,215 bp of chromosome 13). Interestingly, none of the detected loci were found to be significant to either seed protein or oil contents, as well as the eight EAAs. This may indicate that methionine is genetically regulated by unique loci and can be explored to either increase the seed protein content or reduce the pleiotropy between seed protein and oil contents. Phenylalanine was significantly associated with five loci located on chromosomes 2 (8,317,216 bp*, ss715583986*, LOD of 5.3), 5 (40,302,839, *ss715591893*, LOD of 6.6), 19 (38,142,874, *ss715634697*, LOD of 7.0), and 20 (40,704,783, *ss715638104*, LOD of 6.2) (Table 2). Similar to methionine, none of these loci were significantly associated with seed protein and oil contents (Fig. 4). A total of four marker-trait associations across chromosomes 8, 14, and 19 were identified for threonine, with LOD ranging from 5.4 (*ss715635077* located at 41,071,216 bp of chromosome 19) to 8.4 (*ss715633024* located at 1,066,013 bp of chromosome 19). Five unique marker-trait associations were detected across chromosomes 6, 14, 15, 16, and 20 for tryptophan (Fig. 4). LOD scores ranged from 5.6 (*ss715621013* located at 15,735,835 bp of chromosome 15) to 8.1 (*ss715618478* located at 3,556,174 bp of chromosome 14). Lastly, six marker-trait associations across chromosomes 7, 13, 15, and 19 were identified for valine, with LOD ranging from 5.0 (*ss715615305* located at 32,525,645 bp of chromosome 13) to 7.9 (*ss715633024* located at 1,066,013 bp of chromosome 19) (Table 2).

## Discussion

The increase in consumer awareness regarding the socio-environmental impact of food production practices led to substantial growth in the consumption of plant-based protein products^64^. Plant-based protein products provide various environmental benefits compared to animal-derived products, including reduced land, water, and energy requirements. This supports the sustainability of the food production chain, resulting in a lower overall carbon footprint^65^. Hence, the global market for plant-based meat is growing at a fast pace of 14.8% yearly and is estimated to surpass $30 billion by 2026^66^. Soybean protein is a major source of plant-based products accounting for over 70% of global plant-based protein meal for animal feed^2^. Given the importance of seed composition towards soybean seed quality and overall value, breeding programs have emphasized the development of high-yielding cultivars with improved seed composition. However, there are complex interactions among seed components, as well as between seed components and grain yield^18,28,67–72^.

Across 449 genetically diverse PIs used in this study representing most of the genetic diversity among maturity groups 4 to 6, seed protein content was found to be significantly correlated with EAAs (Person’s correlation ranging from 0.283 to 0.985) and seed oil content (− 0.697). It is worth noting that the collected phenotypic data demonstrated high consistency and reliability across environments with Cronbach’s α scores averaging 0.86 across all seed composition traits. The negative phenotypic correlation between seed protein and oil contents has been previously reported in the literature^67,68,72–74^. Although seed protein content may be a good proxy for the EAAs seed concentration, methionine (0.736) and tryptophan (0.283) were found to have a weaker correlation with seed protein content as compared to the remaining EAAs. Similar results have been reported where methionine and tryptophan showed a reduced correlation with seed protein content^67,68,72,75^. In addition, the negative correlation between EAAs and seed oil content (− 0.011 to − 0.673) was shown to be weaker and not proportional to the strength of the correlation between seed protein and oil contents. Tryptophan did not show a significant correlation with seed oil content (− 0.011). The low correlation between tryptophan and both seed protein and oil contents may be attributed to independent biological mechanisms, although the difficulty in measuring this amino acid may also contribute to the observed low correlations^67,68,76^. Hence, these results engage the hypothesis that improving the seed protein content through manipulating the composition of specific EAAs may reduce the degree of pleiotropy between seed protein and oil.

In addition, the interaction between genotype, environment, and management practices (G×E×M) substantially impacts seed protein and oil contents as well as EAAs concentration. For instance, water deficit has been reported to reduce seed protein content while increasing seed oil content^37,77,78^. In contrast, increased precipitation has been associated with reductions in both seed protein and oil contents^79^. Higher temperatures during grain filling have been associated with lower seed protein and higher oil contents^35,37,74,80^, although the relationship between these variables may be dependent on genotype and geographical latitude^35^. Nitrogen fertilization as well as planting dates have also been reported to alter seed protein and oil contents^37,74,81^. To date, however, there are limited reports on the impact of different genetic backgrounds and various seed components on the across-environment stability of seed protein content.

In this study, the across-environment stability of seed protein content was calculated using Shukla’s Stability Variance (*σi*^*2*^)^48^ and ranged from 0.12 (PI 398351) to 17.16 (FC 33243), with an average of 2.70. It indicates that, although environmental parameters and management practices impact seed protein content, genotypes may express different sensitivities to these stimuli. In summary, Shukla’s Stability Variance is a univariate parametric method that estimates the variance (*σ*_*i*_^*2*^) of the genotype by environment interaction (G×E) plus an error term associated with the genotype in multiple environments. Hence, it measures stability rather than performance based on the overall contribution of a genotype to the G×E sums of squares and the stability variance *σ*_*i*_^*2*^*.* A genotype with low *σ*_*i*_^*2*^ shows minimal contribution towards G×E and therefore is considered stable. This methodology has been used to measure yield stability in soybean^82^ and various crops including fava beans (*Vicia faba* L.)^83^, safflower (*Carthamus tinctorius* L.)^84^, winter bread wheat (*Triticum aestivum* L.)^85^, and maize (*Zea mays* L.)^86^. Nevertheless, a limitation of this method is the minimum generalization of measured stability in a different set of tested genotypes. In this study, however, 449 genetically diverse PIs within maturity groups 4–6 were included, which offsets the limited generalization of results since these genotypes cover most of the available genetic diversity within the tested maturity groups.

To further investigate the across-environment seed protein stability, a random forest model was developed using Shukla’s Stability Variance as the response variable and the seed components as the explanatory variables. This approach has been used across multiple disciplines to identify relevant explanatory variables, including selection of molecular markers in genomic prediction^87^, selection of bands derived from hyperspectral data for trait estimation^88^, up to selection of explanatory variables to predict probable biological stream conditions^89^. Herein, explanatory variables had different variable importance towards the observed variation in seed protein stability variance. The EAAs valine (59.24), phenylalanine (40.19), and threonine (33.84) showed the highest Gini Importance across all seed components, whereas both seed protein (20.93) and oil (19.85) contents were among the explanatory variables with the lowest Gini Importance. It indicates that total seed protein and oil contents are not associated with across-environment seed protein stability and that EAAs concentration may partially explain the differences in sensitivity to environmental stimuli in seed protein content across genotypes.

To the best of our knowledge, this is the first report exploring potential underlying causes of G×E affecting across-environment seed protein stability. It unveils opportunities to develop genotypes with more stable across-environment seed protein content by manipulating the EAAs concentration through genetics and breeding. For instance, valine belongs to the branched-chain amino acids (BCAA) and is among the most hydrophobic of amino acids. This property is important for both the stability of folded protein and the folding pathway leading to the mature structure^90,91^. In swine, valine is a great energy source as it is one of the most energy-generating amino acids through the oxidation of branched-chain α-keto acid dehydrogenase complex^92^. Phenylalanine is the precursor of tyrosine^93^. In plants, phenylalanine is essential in the interconnection between primary and secondary metabolism. It is a precursor for numerous plant compounds that are essential for plant reproduction, growth, development, and stress defense^94^. Threonine is one of the most limiting amino acids in swine and poultry diets^95,96^. It is necessary for the synthesis of threonine-rich proteins in epithelial tissues and regulates lipid metabolism, embryonic stem cell proliferation and differentiation, and intestinal health including nutrient digestibility, gut microbiota, intestinal barrier, and immune function^97,98^. Still, further studies are needed to reveal the biological rationale behind the effect of certain EAAs toward across-environment seed protein stability, as well as validate the observed results across environments in various latitudes, as well as a range of genetically diverse genotypes in different maturity groups.

Previous studies have shown the quantitative nature of seed components and the minor effect markers associated with seed protein and oil content as well as EAAs concentration distributed across multiple chromosomes of the soybean genome^27,99–101^. Herein, 56 SNP markers were significantly associated with seed composition traits. Eleven of these SNPs displayed significant associations with two or more traits. Notably, the marker-trait associations found for various seed components across multiple chromosomes unveil their complex genetic architecture and highlight the necessity for dissecting trait interactions and their complex G×E. These results also provide directions for improving the concentration of specific EAAs, as well as reducing the negative correlation between seed protein and oil contents. For instance, while soybean seed contains all EAAs, the concentration of the sulfur-containing EAA methionine is suboptimal for the nutritional needs of monogastric animals^102^. A total of 13 significant marker-trait associations were identified for methionine, of which none have shown significance to seed protein content. This indicates that it is likely possible to improve the seed concentration of methionine through genetics and breeding by targeting genomic regions and genetic resources independent of seed protein. This observation is consistent with previous studies that reported a weaker association between seed protein and methionine^72,75^. In addition, 10 significant marker-trait associations were identified for seed protein while five significant marker-trait associations were identified for seed oil. Interestingly, despite some of these associations occurring in the same chromosome, no overlapping was observed among significant SNPs providing potential molecular directions to uncouple the negative strong correlation between these traits. It is also worth highlighting that, although GWAS has been proven to be a strong tool for identifying significant marker-trait associations and unveiling the genetic architecture of traits of interest, genome-wide prediction models leveraging the genetic correlations among seed components can be explored to improve the identification and selection of genotypes with various desirable traits.

## Conclusions

Manipulating soybean seed components including seed protein and oil content, as well as EAAs concentration, through plant breeding and genetics can maximize the overall value of the crop and its flexibility regarding post-processing applications. However, given the quantitative genetic architecture of such components, several complex interactions observed among seed components and between traits and environmental and management practices substantially challenge the improvement of soybean grain yield and seed components simultaneously. Herein, a thorough investigation of the sensitivity of a wide range of genetically diverse genotypes to environmental stimuli was assessed. It was reported that genotypes may respond differently to such stimuli in regards to seed protein content, and that specific seed components, particularly valine, phenylalanine, and threonine may play a significant role in the overall across-environment protein stability. In addition, various genomic regions have been highlighted to be associated with the variation of multiple seed components. Interestingly, many independent regions (i.e. associated with one or few seed components) were reported. Thus, the manipulation of EAAs through plant breeding and genetics may offset the strong negative correlation between seed protein and oil contents.

### Supplementary Information

Supplementary Table 1.

## Data Availability

The datasets generated and analyzed in this study are available from the corresponding author on reasonable request.

## References

[1] Canella-Vieira C, Chen P (2021). The numbers game of soybean breeding in the United States. Crop Breed. Appl. Biotechnol..

[2] United States Department of Agriculture. *Oilseeds: World Markets and Trade*. https://apps.fas.usda.gov/psdonline/circulars/oilseeds.pdf (2023).

[3] United Soybean Board. *2019 Soybean Meal Demand Assessment*. https://www.unitedsoybean.org/wp-content/uploads/2021/11/2019-Soybean-Meal-Demand-Assessment.pdf (2019).

[4] Bernard JK (2016). Oilseed and oilseed meals. Ref. Module Food Sci..

[5] Wu G (2009). Amino acids: Metabolism, functions, and nutrition. Amino Acids.

[6] Morris CR, Hamilton-Reeves J, Martindale RG, Sarav M, Ochoa-Gautier JB (2017). Acquired amino acid deficiencies: A focus on arginine and glutamine. Nutr. Clin. Pract..

[7] Kuiken KA, Lyman CM (1949). Essential amino acid composition of soybean meals prepared from twenty strains of soy beans. J. Biol. Chem..

[8] Singer, W. M., Zhang, B., Mian, M. A. R. & Huang, H. Soybean amino acids in health, genetics, and evaluation. In *Soybean for Human Consumption and Animal Feed* (ed. Sudarić, A.) Ch. 2 (IntechOpen, 2019). 10.5772/intechopen.89497.

[9] Fernandez SR, Aoyagi S, Han Y, Parsons CM, Baker DH (1994). Limiting order of amino acids in corn and soybean meal for growth of the chick. Poult. Sci..

[10] Bornstein S, Lipstein B (1975). The replacement of some of the soybean meal by the first limiting amino acids in practical broiler diets. Br. Poult. Sci..

[11] Warnick RE, Anderson JO (1968). Limiting essential amino acids in soybean meal for growing chickens and the effects of heat upon availability of the essential amino acids. Poult. Sci..

[12] George AA, De Lumen BO (1991). A novel methionine-rich protein in soybean seed: Identification, amino acid composition, and N-terminal sequence. J. Agric. Food Chem..

[13] Clarke EJ, Wiseman J (2000). Developments in plant breeding for improved nutritional quality of soya beans I. Protein and amino acid content. J. Agric. Sci..

[14] Bellaloui N, Mengistu A (2008). Seed composition is influenced by irrigation regimes and cultivar differences in soybean. Irrig. Sci..

[15] Bellaloui N, Abbas HK, Gillen AM, Abel CA (2009). Effect of glyphosate−boron application on seed composition and nitrogen metabolism in glyphosate-resistant soybean. J. Agric. Food Chem..

[16] Friedman M, Brandon DL (2001). Nutritional and health benefits of soy proteins. J. Agric. Food Chem..

[17] Cromwell GL (1999). Variability among sources and laboratories in nutrient analyses of corn and soybean meal. J. Anim. Sci..

[18] Assefa Y (2019). Assessing variation in US soybean seed composition (protein and oil). Front. Plant Sci..

[19] de Borja Reis AF (2020). Historical trend on seed amino acid concentration does not follow protein changes in soybeans. Sci. Rep..

[20] Kambhampati S (2019). On the inverse correlation of protein and oil: Examining the effects of altered central carbon metabolism on seed composition using soybean fast neutron mutants. Metabolites.

[21] Diers BW, Keim P, Fehr WR, Shoemaker RC (1992). RFLP analysis of soybean seed protein and oil content. Theor. Appl. Genet..

[22] Wang J (2015). Identification and mapping of stable QTL for protein content in soybean seeds. Mol.r Breed..

[23] Vaughn JN, Nelson RL, Song Q, Cregan PB, Li Z (2014). The genetic architecture of seed composition in soybean is refined by genome-wide association scans across multiple populations. Genes Genomes Genetics.

[24] Hwang E-Y (2014). A genome-wide association study of seed protein and oil content in soybean. BMC Genom..

[25] Bolon Y-T (2010). Complementary genetic and genomic approaches help characterize the linkage group I seed protein QTL in soybean. BMC Plant Biol..

[26] Jun T-H, Van K, Kim MY, Lee S-H, Walker DR (2008). Association analysis using SSR markers to find QTL for seed protein content in soybean. Euphytica.

[27] Bandillo N (2015). A population structure and genome-wide association analysis on the USDA soybean germplasm collection. Plant Genome.

[28] Patil G (2017). Molecular mapping and genomics of soybean seed protein: A review and perspective for the future. Theor. Appl. Genet..

[29] Lee J-D, Shannon JG, Choung M-G (2010). Selection for protein content in soybean from single F2 seed by near infrared reflectance spectroscopy. Euphytica.

[30] Shorter R, Byth D, Mungomery V (1977). Estimates of selection parameters associated with protein and oil content of soybean seeds (*Glycine max* (L.) Merr.). Aust. J. Agric. Res..

[31] Kumar V, Rani A, Solanki S, Hussain SM (2006). Influence of growing environment on the biochemical composition and physical characteristics of soybean seed. J. Food Compos. Anal..

[32] Medic J, Atkinson C, Hurburgh CR (2014). Current knowledge in soybean composition. J. Am. Oil Chem. Soc..

[33] Thomas JMG, Boote KJ, Allen LH, Gallo-Meagher M, Davis JM (2003). Elevated temperature and carbon dioxide effects on soybean seed composition and transcript abundance. Crop Sci..

[34] Wolf RB, Cavins JF, Kleiman R, Black LT (1982). Effect of temperature on soybean seed constituents: Oil, protein, moisture, fatty acids, amino acids and sugars. J. Am. Oil Chem. Soc..

[35] Piper EL, Boote KI (1999). Temperature and cultivar effects on soybean seed oil and protein concentrations. J. Am. Oil Chem. Soc..

[36] Vollmann J, Fritz CN, Wagentristl H, Ruckenbauer P (2000). Environmental and genetic variation of soybean seed protein content under Central European growing conditions. J. Sci. Food Agric..

[37] Rotundo JL, Westgate ME (2009). Meta-analysis of environmental effects on soybean seed composition. Field Crops Res..

[38] Tabe L, Hagan N, Higgins TJV (2002). Plasticity of seed protein composition in response to nitrogen and sulfur availability. Curr. Opin. Plant Biol..

[39] Song Q (2013). Development and evaluation of SoySNP50K, a high-density genotyping array for soybean. PLoS One.

[40] Song Q (2015). Fingerprinting soybean germplasm and its utility in genomic research. Genes Genomes Genetics.

[41] Fehr, W. R. & Caviness, C. E. Stages of soybean development (Special report 80). Iowa State University. https://dr.lib.iastate.edu/handle/20.500.12876/90239 (1977).

[42] Chen P, Sneller CH, Mozzoni LA, Rupe JC (2007). Registration of ‘Osage’ soybean. J. Plant Regist..

[43] R Core Team. R: A language and environment for statistical computing. http://www.r-project.org/ (2023).

[44] Kuznetsova A, Brockhoff PB, Christensen RHB (2017). lmerTest package: Tests in linear mixed effects models. J. Stat. Softw..

[45] Bates D, Mächler M, Bolker BM, Walker SC (2015). Fitting linear mixed-effects models using lme4. J. Stat. Softw..

[46] Cronbach LJ (1951). Coefficient alpha and the internal structure of tests. Psychometrika.

[47] Revelle, W. *psych: Procedures for Psychological, Psychometric, and Personality Research* 1–457 (Springer, 2021).

[48] Shukla GK (1972). Some statistical aspects of partitioning genotype-environmental components of variability. Heredity (Edinb.).

[49] van Rossum, B. *statgenGxE: Genotype by Environment (GxE) Analysis*. R package version 1.0.7 (2024).

[50] Domitruk DR, Duggan BL, Fowler DB (2001). Genotype–environment interaction of no-till winter wheat in Western Canada. Can. J. Plant Sci..

[51] Lin CS, Binns MR, Lefkovitch LP (1986). Stability analysis: Where do we stand?. Crop Sci..

[52] Breiman L (2001). Random forests. Mach. Learn..

[53] Menze BH (2009). A comparison of random forest and its Gini importance with standard chemometric methods for the feature selection and classification of spectral data. BMC Bioinform..

[54] Kuhn M (2008). Building predictive models in R using the caret package. J. Stat. Softw..

[55] Huang M, Liu X, Zhou Y, Summers RM, Zhang Z (2019). BLINK: A package for the next level of genome-wide association studies with both individuals and markers in the millions. Gigascience.

[56] Lipka AE (2012). GAPIT: Genome association and prediction integrated tool. Bioinformatics.

[57] Liu X, Huang M, Fan B, Buckler ES, Zhang Z (2016). Iterative usage of fixed and random effect models for powerful and efficient genome-wide association studies. PLoS Genet..

[58] Amirrudin M, Nasution K, Supahar S (2020). Effect of variability on cronbach alpha reliability in research practice. J. Mate. Stat. Komputasi.

[59] Bland JM, Altman DG (1997). Statistics notes: Cronbach’s alpha. BMJ.

[60] Canella-Vieira C (2022). Differential responses of soybean genotypes to off-target dicamba damage. Crop Sci..

[61] Canella Vieira C (2022). Identification of genomic regions associated with soybean responses to off-target dicamba exposure. Front. Plant Sci..

[62] Tavakol M, Dennick R (2011). Making sense of Cronbach’s alpha. Int. J. Med. Educ..

[63] Bernardo R (2020). Reinventing quantitative genetics for plant breeding: Something old, something new, something borrowed, something BLUE. Heredity (Edinb.).

[64] Hwang J, You J, Moon J, Jeong J (2020). Factors affecting consumers’ alternative meats buying intentions: Plant-based meat alternative and cultured meat. Sustainability.

[65] Wang Y, Cai W, Li L, Gao Y, Lai K (2023). Recent advances in the processing and manufacturing of plant-based meat. J. Agric. Food Chem..

[66] Watson, J. Plant-based Meat Market To Reach USD 30.92 Billion By 2026|Reports And Data. *Reports and Data*. https://www.globenewswire.com/news-release/2019/10/14/1929284/0/en/Plant-based-Meat-Market-To-Reach-USD-30-92-Billion-By-2026-Reports-And-Data.html/ (2019).

[67] Pfarr MD, Kazula MJ, Miller-Garvin JE, Naeve SL (2018). Amino acid balance is affected by protein concentration in soybean. Crop Sci..

[68] Borja-Reis AF (2020). Historical trend on seed amino acid concentration does not follow protein changes in soybeans. Sci. Rep..

[69] Morrison MJ, Voldeng HD, Cober ER (2000). Agronomic changes from 58 years of genetic improvement of short-season soybean cultivars in Canada. Agron. J..

[70] Ortez OA (2018). Exploring nitrogen limitation for historical and modern soybean genotypes. Agron. J..

[71] Mahmoud AA (2006). Effect of six decades of selective breeding on soybean protein composition and quality: A biochemical and molecular analysis. J. Agric. Food Chem..

[72] Wilcox JR, Shibles RM (2001). Interrelationships among seed quality attributes in soybean. Crop Sci..

[73] Hymowitz T, Collins FI, Panczner J, Walker WM (1972). Relationship between the content of oil, protein, and sugar in soybean seed. Agron. J..

[74] Mourtzinis S, Gaspar AP, Naeve SL, Conley SP (2017). Planting date, maturity, and temperature effects on soybean seed yield and composition. Agron. J..

[75] Burton JW, Purcell AE, Walter WM (1982). Methionine concentration in soybean protein from populations selected for increased percent protein. Crop Sci..

[76] Zarkadas CG (2007). Assessment of the protein quality of fourteen soybean [*Glycine max* (L.) Merr.] cultivars using amino acid analysis and two-dimensional electrophoresis. Food Res. Int..

[77] Šarčević H (2022). Stability of protein and oil content in soybean across dry and normal environments—a case study in croatia. Agronomy.

[78] Carrera C, Martínez MJ, Dardanelli J, Balzarini M (2009). Water deficit effect on the relationship between temperature during the seed fill period and soybean seed oil and protein concentrations. Crop Sci..

[79] Maestri DM (1998). Seed composition of soybean cultivars evaluated in different environmental regions. J. Sci. Food Agric..

[80] Kane MV, Steele CC, Grabau LJ, MacKown CT, Hildebrand DF (1997). Early-maturing soybean cropping system: III. Protein and oil contents and oil composition. Agron. J..

[81] Ortel CC (2020). Soybean maturity group and planting date influence grain yield and nitrogen dynamics. Agrosyst. Geosci. Env..

[82] Döttinger CA, Hahn V, Leiser WL, Würschum T (2023). Do we need to breed for regional adaptation in soybean?—Evaluation of genotype-by-location interaction and trait stability of soybean in Germany. Plants.

[83] Temesgen T, Keneni G, Sefera T, Jarso M (2015). Yield stability and relationships among stability parameters in faba bean (*Vicia faba* L.) genotypes. Crop J..

[84] Afzal O, Hassan F, Ahmed M, Shabbir G, Ahmed S (2021). Determination of stable safflower genotypes in variable environments by parametric and non-parametric methods. J. Agric. Food Res..

[85] Roostaei M (2022). Genotype × environment interaction and stability analyses of grain yield in rainfed winter bread wheat. Exp. Agric..

[86] Changizi M, Choukan R, Heravan EM, Bihamta MR, Darvish F (2014). Evaluation of genotype×environment interaction and stability of corn hybrids and relationship among univariate parametric methods. Can. J. Plant Sci..

[87] Montesinos López, O. A., Montesinos López, A. & Crossa, J. Random forest for genomic prediction. In *Multivariate Statistical Machine Learning Methods for Genomic Prediction* 633–681 (Springer International Publishing, 2022). 10.1007/978-3-030-89010-0_15. 36103587

[88] Abdel-Rahman EM, Ahmed FB, Ismail R (2013). Random forest regression and spectral band selection for estimating sugarcane leaf nitrogen concentration using EO-1 Hyperion hyperspectral data. Int. J. Remote Sens..

[89] Fox EW (2017). Assessing the accuracy and stability of variable selection methods for random forest modeling in ecology. Environ. Monit. Assess..

[90] Brosnan JT, Brosnan ME (2006). Branched-chain amino acids: Enzyme and substrate regulation. J. Nutr..

[91] Zhu B, Zhou ME, Kay CM, Hodges RS (1993). Packing and hydrophobicity effects on protein folding and stability: Effects of β-branched amino acids, valine and isoleucine, on the formation and stability of two-stranded α-helical coiled coils/leucine zippers. Protein Sci..

[92] Shimomura Y, Harris RA (2006). Metabolism and physiological function of branched-chain amino acids: Discussion of session 1. J. Nutr..

[93] Kohlmeier, M. Phenylalanine. In *Nutrient Metabolism* 314–321 (Elsevier, 2003). 10.1016/B978-012417762-8.50051-X.

[94] Pascual MB (2016). Biosynthesis and metabolic fate of phenylalanine in conifers. Front. Plant Sci..

[95] Karau, A. & Grayson, I. Amino acids in human and animal nutrition. In *Biotechnology of Food and Feed Additives* (eds. Zorn, H. & Czermak, P.) 189–228 (Springer Link, 2014). 10.1007/10_2014_269. 10.1007/10_2014_269 24676880

[96] Chen YP (2017). Effects of threonine supplementation on the growth performance, immunity, oxidative status, intestinal integrity, and barrier function of broilers at the early age. Poult. Sci..

[97] Tang Q, Tan P, Ma N, Ma X (2021). Physiological functions of threonine in animals: Beyond nutrition metabolism. Nutrients.

[98] Wang X (2007). A deficiency or excess of dietary threonine reduces protein synthesis in jejunum and skeletal muscle of young pigs. J. Nutr..

[99] Zhang J (2018). Genome-wide scan for seed composition provides insights into soybean quality improvement and the impacts of domestication and breeding. Mol. Plant.

[100] Panthee DR, Pantalone VR, West DR, Saxton AM, Sams CE (2005). Quantitative trait loci for seed protein and oil concentration, and seed size in soybean. Crop Sci..

[101] Mao T (2013). Identification of quantitative trait loci underlying seed protein and oil contents of soybean across multi-genetic backgrounds and environments. Plant Breed..

[102] Krishnan HB, Jez JM (2018). Review: The promise and limits for enhancing sulfur-containing amino acid content of soybean seed. Plant Sci..

